# Statistical analysis plan for the Erythropoietin in Traumatic Brain Injury trial: a randomised controlled trial of erythropoietin versus placebo in moderate and severe traumatic brain injury

**DOI:** 10.1186/1745-6215-15-501

**Published:** 2014-12-20

**Authors:** Jeffrey Presneill, Lorraine Little, Alistair Nichol, Craig French, D James Cooper, Samir Haddad, Jacques Duranteau, Olivier Huet, Markus Skrifvars, Yaseen Arabi, Rinaldo Bellomo, on behalf of the EPO-TBI Investigators and the ANZICS Clinical Trials Group

**Affiliations:** Australian and New Zealand Intensive Care Research Centre, Monash University School of Public Health and Preventive Medicine, 99 Commercial Road, Melbourne, 3004 Australia; Department of Intensive Care, Mater Health Services, Raymond Terrace, South Brisbane, 4101 Australia; Department of Anaesthesia and Intensive Care Medicine, St Vincent’s University Hospital, Elm Park, Dublin 4, Ireland; School of Medicine and Medical Sciences, University College, Elm Park, Dublin 4, Ireland; Department of Intensive Care Medicine, The Alfred, Commercial Road, Melbourne, 3004 Australia; Department of Intensive Care, Western Health, Gordon Street, Footscray, 3011 Australia; The University of Melbourne, Grattan Street, Parkville, 3052 Australia; Intensive Care Department, King Abdulaziz Medical City, PO Box 22490, Riyadh, 11426 Kingdom of Saudi Arabia; Service d’Anesthésie-Réanimation, Hôpitaux universitaires Paris Sud, Assistance Publique des Hôpitaux de Paris, Hôpital de Bicêtre, 78, rue du Général Leclerc, Paris, 94275 Le Kremlin Bicêtre France; Department of Anesthesiology and Intensive Care Medicine, CHRU La Cavale Blanche Université de Bretagne Ouest, Brest, Cedex, 29609 France; Department of Anaesthesiology and Intensive Care Medicine, Helsinki University Hospital, P.O. Box 266, Helsinki, FIN-00029 Finland; Intensive Care Department, College of Medicine King Saud Bin Abdulaziz University for Health Sciences and King Abdullah International Medical Research Center, PO Box 22490, Riyadh, 11426 Kingdom of Saudi Arabia; Department of Intensive Care, Austin Health, Studley Road, Heidelberg, 3084 Australia

**Keywords:** Traumatic brain injury, Erythropoietin, Outcome, Critical care, Randomised controlled trials

## Abstract

**Background:**

The Erythropoietin in Traumatic Brain Injury (EPO-TBI) trial aims to determine whether the administration of erythropoietin to patients with moderate or severe traumatic brain injury improves patient-centred outcomes.

**Methods:**

EPO-TBI is a multicentre, blinded, randomised, parallel groups, placebo-controlled, phase III superiority trial of erythropoietin in ICU patients with traumatic brain injury conducted in Australia and New Zealand, Saudi Arabia and Europe; 606 critically ill patients aged 15 to 65 years with moderate or severe acute traumatic brain injury will be enrolled.

Trial patients will receive either 40,000 IU erythropoietin or placebo by subcutaneous injection administered weekly for up to three doses during their ICU admission.

The primary outcome measure is the proportion of unfavourable neurological outcomes, comprising death or severe disability, observed at 6 months following randomisation utilizing the Extended Glasgow Outcome Scale. Secondary outcomes, also assessed at 6 months following randomisation, include the probability of an equal or greater Extended Glasgow Outcome Scale level, mortality, the proportions of patients with proximal deep venous thrombosis or with composite thrombotic vascular events, as well as assessment of quality of life and cost-effectiveness. The planned sample size will allow 90% power to detect a reduction from 50% to 36% in unfavourable neurological outcomes at a two-sided alpha of 0.05.

**Discussion:**

A detailed analysis plan has been developed for EPO-TBI that is consistent with international guidelines. This plan specifies the statistical models for evaluation of primary and secondary outcomes, as well as defining covariates for adjusted analyses.

Application of this statistical analysis plan to the forthcoming EPO-TBI trial will facilitate unbiased analyses of these important clinical data.

**Trial registration:**

Australian New Zealand Clinical Trials Registry: ACTRN12609000827235 (22 September 2009). ClinicalTrials.gov: NCT00987454 (29 September 2009). European Drug Regulatory Authorities Clinical Trials: 2011-005235-22 (18 January 2012).

**Electronic supplementary material:**

The online version of this article (doi:10.1186/1745-6215-15-501) contains supplementary material, which is available to authorized users.

## Update

The international multicentre Erythropoietin in Traumatic Brain Injury (EPO-TBI) trial commenced in May 2010, with patient recruitment concluding in November 2014 and final collection of all 6-month outcome data scheduled by May 2015. A manuscript describing the trial protocol (Nichol and colleagues) was submitted to *Trials* in September 2014. The following statistical analysis plan, determined prior to final trial data availability, specifies the statistical models for evaluation of the EPO-TBI primary and secondary outcomes, including definition of the covariates to be included in various adjusted analyses.

## Introduction

Traumatic brain injury (TBI) is a leading cause of mortality and long-term disability, particularly affecting young people. Even a small increase in the number of TBI victims who are able to live independently, instead of being permanently disabled, would yield major human and economic gains [1, 2].

Erythropoietin (EPO) has shown promise as an agent to attenuate TBI, as EPO appears to have neuroprotective effects as well as its better known effect on erythropoiesis [3]. EPO-TBI is an international multicentre, blinded, randomised, parallel groups, placebo-controlled, phase III superiority trial of erythropoietin administration to adult ICU patients with acute TBI of at least moderate severity [4, 5].

The trial is being conducted, and accumulating data monitored, according to the standard requirements of Good Clinical Practice [6]. Ethics and regulatory approvals of the protocol and related documents were obtained prior to commencing the trial at each site according to state or national legislation. The list of responsible ethics committees is provided in Additional file 1.

Informed consent was obtained from each patient’s legal surrogate for participation in the trial. Patients who recover sufficient cognition to understand the explanation of the trial will additionally be asked to consent to continue in the trial if this is required under the ethics committee approval conditions. In France patients may be enrolled under the Emergency clause. More details of the trial protocol and participating hospitals are available from the trial registration site [7] as well as from a forthcoming manuscript (Nichol and colleagues, manuscript submitted).

General confidence in the final results and conclusions of clinical trials is enhanced when the statistical approaches to outcome analyses are specified prior to the availability of trial data. The following statistical analysis plan complies with recommendations for the Consolidated Standards of Reporting Trials [8] as well as guidance from the International Conference on Harmonisation of Technical Requirements for Registration of Pharmaceuticals for Human Use, especially “Statistical principles for clinical trials E9” [9] and “Structure and content of clinical study reports E3” [10].

This statistical analysis plan identifies the procedures to be applied to the primary and secondary outcome analyses in the whole trial cohort once trial data validation is complete. Covariates for adjusted analyses and selected subgroups of interest are also pre-specified.

## Methods

### Study design and definitions

EPO-TBI is a multicentre, prospective, two parallel groups, blinded, placebo-controlled, randomised phase III superiority trial of erythropoietin administration to adult ICU patients with TBI of at least moderate severity. The primary objective of this trial is to demonstrate that long-term neurological function assessed 6 months after injury is improved following early administration of EPO compared to a placebo control. Exploratory secondary and adjusted multivariable analyses will also be conducted.

### Trial population and eligibility

A total of 606 TBI patients will be enrolled in Australia, New Zealand, Saudi Arabia, France, Finland, Germany, and Ireland. There are 29 individual research sites defined by the hospital treating each patient, with investigators representing a team of clinicians recruiting patients at one or several related hospitals. The inclusion and exclusion criteria are presented in Table 1. Eligible patients will be randomised to receive by subcutaneous injection either erythropoietin (Epoetin alfa, Jansen-Cilag Pty Ltd; 40,000 IU in 1 mL from a pre-filled syringe) or an equal volume placebo comprising 0.9% sodium chloride. The study drug or placebo will be administered once per week for up to three doses provided the patient continues to require ICU care and meets no drug-withholding criteria. Measurements of treatment compliance will consist of summaries of the count of EPO/placebo doses received.

**Table 1:** **Inclusion and exclusion criteria for the Erythropoietin in Traumatic Brain Injury (EPO-TBI) trial**

Inclusion	Patients with non-penetrating moderate (Glasgow Coma Score (GCS) 9–12) or severe (GCS 3–8) traumatic brain injury admitted to an ICU who:
	1. Are ≥15 to ≤65 years of age*
	2. Are <24 hours since primary traumatic injury
	3. Are expected to stay ≥48 hours
	4. Have a haemoglobin not exceeding the upper limit of the applicable normal reference range in clinical use at the treating institution**
	5. Have written informed consent from legal surrogate
**Exclusion**	Patients are excluded from the study if any of the following criteria apply^#^:
	1. GCS = 3 and fixed dilated pupils
	2. History of deep vein thrombosis, pulmonary embolism or other thromboembolic event
	3. A chronic hypercoagulable disorder, including known malignancy
	4. Treatment with erythropoietin in the last 30 days
	5. First dose of study drug unable to be given within 24 hours of primary injury
	6. Pregnancy or lactation or 3 months post-partum
	7. Uncontrolled hypertension (systolic blood pressure >200 mmHg or diastolic blood pressure >110 mmHg)
	8. Acute myocardial infarct within the past 12 months
	9. Past history of epilepsy with seizures in past 3 months
	10. Expected to die imminently (<24 hours)
	11. Inability to perform lower limb ultrasounds
	12. Known sensitivity to mammalian cell-derived products
	13. Hypersensitivity to the active substance or to any of the additives
	14. Pure red cell aplasia
	15. End-stage renal failure (receives chronic dialysis)
	16. Severe pre-existing physical or mental disability or severe co-morbidity that may interfere with the assessment of outcome
	17. Spinal cord injury
	18. Treatment with any investigational drug within 30 days before enrolment
	19. The treating physician believes it is not in the best interests of the patient to be randomised to this trial

*6 sites had minimum age 15 years, 13 sites minimum age 16 years and 10 sites minimum age 18 years.

**< 140 g/L at Johannes Gutenberg-Universtität, Mainz Germany.

**<148 g/L for males and < 135 g/L for females at Royal Adelaide Hospital, Adelaide Australia.

^#^Additional exclusion criteria at Johannes Gutenberg-Universtität, Mainz Germany. Uncontrolled hypertension (systolic blood pressure >160 mm Hg or diastolic blood pressure >90 mm Hg), morbid obesity, coronary artery disease, peripheral arterial occlusive disease, vascular disease of the carotid arteries, cerebrovascular disorders, recent stroke, contraindications against prophylaxis of DVT or an increased risk for DVT (e.g. with additional trauma and/or operations, severe varicose veins, severe smokers, intake of oral contraceptives, infections and inflammation).

### Randomisation

Trial treatment is allocated between EPO and placebo in a ratio of 1:1 via a confidential internet-based 24 hour centralised computer-generated randomisation schedule which provides concealed immediate assignment. Balance in treatment allocation across the study participants will be enhanced through stratification by both research site (defined by each participating hospital) as well as by each patient’s initial pre-intubation Glasgow Coma Score (GCS); with a score of 9 to 12 indicating moderate, and a GCS of 3 to 8 indicating severe TBI. Randomisation within these strata will follow a permuted block scheme to further enhanced treatment balance.

### Treatment masking (blinding)

The trial will be conducted as a blinded trial. Patients, site investigators, site research coordinators, the French management team, staff at the Australian and New Zealand Intensive Care Research Centre (ANZIC-RC), central outcome assessors (Appendix 1), and the statisticians for interim and final analyses will not be advised of treatment allocation.

For the efficient conduct of the trial several staff will be unblinded. They are the research site pharmacist, the central pharmacy in France (Clinical Trial Department of the Pharmaceutical Establishment of Assistance Publique-Hôpitaux de Paris), the ANZIC-RC unblinded project officer (Appendix 2), a nominated statistician who will supervise data extraction from the database for interim and final analyses, and the Monash University Clinical Informatics and Data Management Unit [11] staff who administer the internet-based computerised randomisation process. Also, for reasons of safety, unblinded dosing nurses will be allocated in each research site to administer EPO or placebo doses discreetly with a screen around the patient bed area. The trial drug/placebo dose may be checked with a second unblinded nurse if required to comply with local hospital regulations. These unblinded dosing nurses have access to the unblinded trial pharmacist. Unblinded dosing nurses will not be involved in the care of a trial patient and may not discuss study drug treatment with research staff or other members of the ICU or hospital staff.

When final database entries have been made and final queries have been resolved, the EPO-TBI research database will be locked. Application of this trial statistical analysis plan to the computation of treatment effect estimates will then proceed with a second, independent statistician using a blinded binary indicator of treatment to generate primary and secondary effect estimates. These estimates will be incorporated in the trial final report by the trial’s writing committee while unaware of the treatment code.

### Study objectives and endpoints

**Figure 1:**
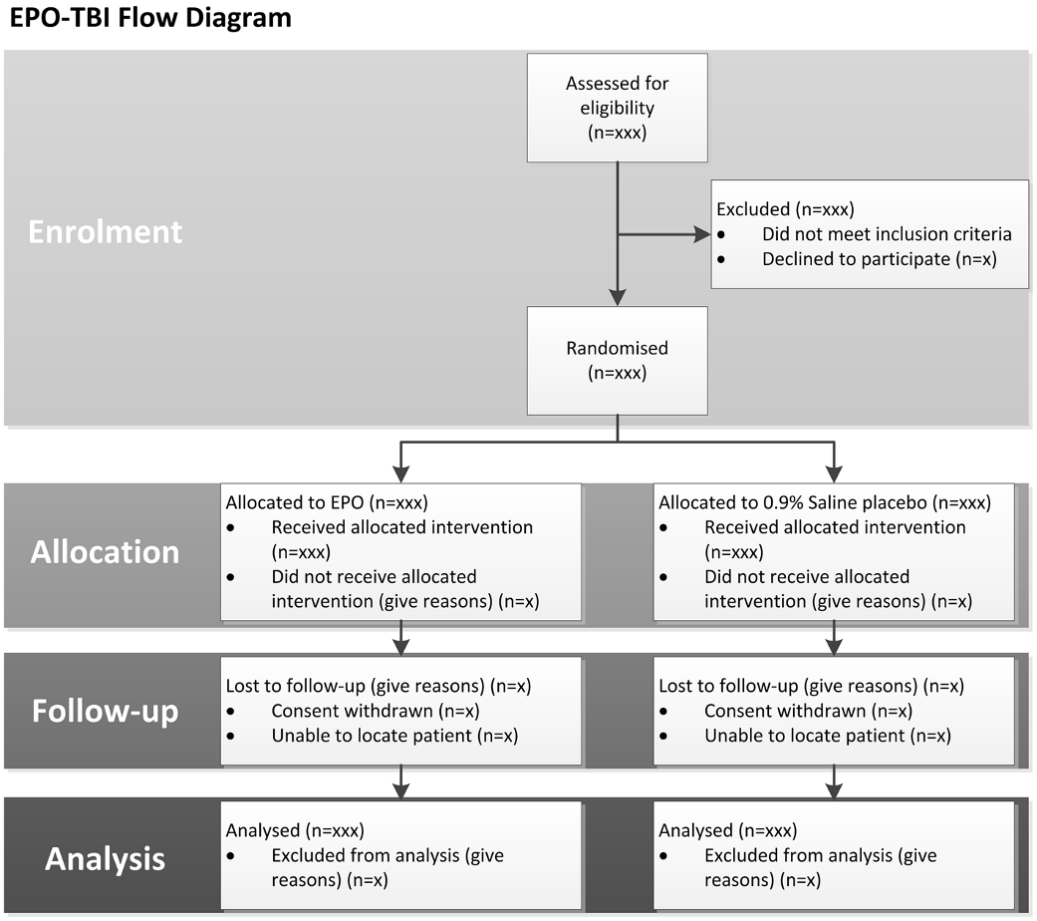
**CONSORT flow diagram.**

A CONSORT flow diagram will be generated as shown in Figure 1.

Although the trial will be analysed as a whole, demographic, baseline, and post-baseline data, as well as primary and secondary outcomes, will also be tabulated by centre and region and/or presented graphically according to randomised treatment and summarised using descriptive statistics.

No imputation for missing data will be performed and the number of analysed observations will be reported for each summary proportion. The main primary and secondary analyses will follow a modified intention-to-treat approach to define the full analysis patient set, based on all randomly assigned patients except those withdrawing consent for use of all trial data and those not fulfilling inclusion criteria and never receiving the intervention [12].

#### Primary outcome

The primary outcome will be the proportion of patients with unfavourable neurological outcomes at 6 months from randomisation. This is defined from a mid-point dichotomization of the eight level ordinal Extended Glasgow Outcome Scale (GOSE) [13], with unfavourable outcomes comprising severe disability (GOSE 2 to 4), or death (GOSE 1).

The primary outcome will be modelled as a binomial random variable, with a null hypothesis of equality between EPO and placebo groups in the proportion of subjects with an unfavourable outcome. This will be assessed with an uncorrected chi-square test applied to the 2 × 2 contingency table comprising the full analysis set of patients according to randomised treatment group. This primary trial outcome will be reported as an unadjusted risk ratio with associated 95% confidence interval (CI) and also as the risk difference with 95% CI. The number needed to treat for benefit or harm will also be reported if a statistically significant difference between treatment groups is demonstrated. The odds ratio and 95% CI calculated for the primary outcome will be reported in a table of supplementary results.

#### Secondary outcomes and pre-specified covariates

Secondary outcome measures are:

i. Probability of an equal or greater GOSE level at 6 months compared to the probability of a lesser GOSE level
ii. Mortality at 6 months
iii. Proportion of patients with proximal deep venous thrombosis (DVT) detected during regular screening by compression Doppler ultrasound
iv. Proportion of patients with composite thrombotic vascular events (DVT, pulmonary embolism, myocardial infarction, cardiac arrest and cerebrovascular events) at 6 months
v. Quality of life assessment (assessed by two standardized questionnaires: Short Form-12 (SF-12) and EuroQol five dimensions (EQ-5D)) at 6 months

Sensitivity analyses of the primary and secondary outcomes will be performed using logistic regression adjusting for pre-specified baseline covariates as well as any covariate exhibiting substantial imbalance between randomisation arms, as recommended [14]. Baseline variables to be included as fixed effects when developing adjusted outcomes models comprise the following:

i. Geographic region (Australia combined with New Zealand, Saudi Arabia, Europe)
ii. Age
iii. GCS
iv. Pupil reactivity
v. Hypoxia
vi. Hypotension
vii. Marshall computerised tomography brain scan classification [15]

A proportional odds cumulative logit model [16, 17], adjusting for the same covariates, will be applied to the eight-level vector of 6-month GOSE.

A further series of exploratory secondary analyses will fit logistic models including only two covariates, namely EPO treatment and the extended IMPACT TBI probability of 6 month unfavourable outcome [18]. IMPACT extended is a composite score comprising many of the above listed baseline variables.

Adjusted estimates of the effect of EPO derived from logistic and proportional odds ordinal logistic models will be reported as adjusted risk ratios averaged over the remaining covariates, as recently recommended [19–21]. The corresponding odds ratios with 95% CIs will be reported in supplementary tables.

Log binomial regression models will be applied to return adjusted risk ratio estimates incorporating the same fixed effects covariate set as logistic models while also incorporating as a random effect the multiple levels of stratification.

Other secondary analyses also will compare treatment groups using unadjusted and adjusted logistic regression or log binomial regression as above, including assessment of outcomes according to actual treatment received (per-protocol analysis), mortality at hospital discharge and 6 months, and the proportions of adverse events including lower limb DVT or a defined composite adverse thrombotic event outcome.

Time-to-event analyses, including death, the first development of a proximal lower limb DVT or a composite thrombotic complication, will be undertaken using Kaplan-Meier curves assessed by log-rank tests, as well as unadjusted and adjusted Cox proportional hazard regression models returning hazard ratios with 95% CI.

Quality of life outcomes will be reported as means (with standard deviations) of the physical and mental health scores of the SF-12 [22], and as the proportion of reported health problems for each domain of the EQ-5D-3 L [23]. Differences in quality of life between groups will be assessed using two-sample *t* tests or Wilcoxon rank-sum tests as appropriate for the SF-12 component scores, and Fisher exact or chi-squared tests for the proportions in each domain of the EQ-5D-3 L.

Cost-effectiveness will be calculated as a cost per additional patient with a favourable neurological outcome at 6 months following randomisation (defined as GOSE 5–8) and the cost per additional quality-adjusted life year, with quality-adjusted life years calculated using utility scores derived from the EQ-5D-3 L conducted at 6 months post-randomisation. Costs will be determined based on resource use during the intensive care, acute and post-acute periods up to 6 months post-randomisation, and will include costs of EPO where appropriate.

#### Planned subgroups to assess interactions with erythropoietin therapy

Assessment for differential EPO effects across two subgroups will be obtained using interaction terms in logistic regression models. These subgroups are defined by the presence or absence of: 1) severe TBI (GCS 3 to 8); and 2) an intracranial mass lesion according to the Marshall computerised tomography scan classification (V or VI).

#### Data monitoring, interim analyses and statistical significance

An independent Data and Safety Monitoring Committee (DSMC) conducted oversight of the quality of the trial and had access to trial outcome and accumulated safety data, including the differential proportions of DVT and total mortality.

A group sequential statistical approach was used to perform two equally spaced interim analyses (at 33% and 66% of total recruitment) to assess the trial primary outcome. There was no provision to stop early for futility. The Haybittle-Peto criterion (|Z_k_| ≥ 3) for early stopping [24] was applied at these first and second interim analyses by the DSMC, and on both occasions no early stopping was necessary. Because of the negligible effect of the two interim analyses on expenditure of error (final critical value |Z_3_| ≥ 1.975 (*P* value 0.048), rather than 1.960), the final analyses at full recruitment will be little affected by these interim analyses and consequently all final analyses will be conducted with a Type I error alpha equal to 0.05. This level of significance will not be adjusted for multiplicity; however, the primary trial outcome is clearly defined. Unless otherwise specified, all hypothesis tests and accompanying significance levels (that is, *P* values) will be two-sided, with 95% CI.

### Sample size and power

The trial sample size was calculated for the primary outcome using an estimated baseline 50% proportion of unfavourable neurological outcomes (death and severe disability) at 6 months from randomisation [2, 25]. Inclusion of 574 evaluable patients (two equally sized groups of 287 subjects) was estimated to provide more than 90% power to detect a 28% relative risk reduction (from 50% to 36%), and approximately 80% power to detect a 24% relative risk reduction (from 50% to 38%), in unfavourable neurological outcomes at a two-sided Type I alpha error of 0.05. A trial of this size also was estimated to have an 80% power to detect a 9% absolute risk increase in proximal lower limb DVT from an assumed baseline proportion of 18% (50% increase in relative risk) at a one-sided alpha of 0.05. The target sample size was inflated from 574 to 606 patients to allow for a combined withdrawal and loss to follow-up proportion of 5%.

#### Model fitting and goodness of fit assessment

Derivation of final parsimonious multivariable models and assessment of their fit to the trial data will follow standard approaches [16, 26], including by evaluation of likelihood ratio tests to compare nested models, and otherwise each model’s Akaike Information Criterion. Evaluation of model fit will include assessment of the link function and the Hosmer-Lemeshow goodness-of-fit statistic [16].

Proportionality in ordinal logistic regression models will be assessed [27]. Also, the proportional hazards assumption across treatment arms in time-to-event analyses will be evaluated using scaled Schoenfeld residuals [28] and visual assessment of log-log plots.

### Analysis software

Data capture and processing occurs initially at Monash University Clinical Informatics and Data Management Unit [11], and these data will be exported in relevant formats for statistical analysis using current versions of SAS (SAS Institute Inc. Cary, NC, USA) and Stata (StataCorp LP, College Station, Texas, USA).

#### Safety and adverse event analyses

Safety and tolerability implications will be summarized using descriptive statistical methods, supplemented by calculation of CIs where appropriate. Patients with protocol deviations, adverse events and missing values will be identified, and a descriptive analysis undertaken including their relationship to treatment.

The known risk of thrombosis with EPO therapy will be specifically assessed by two secondary outcomes of this trial, and compliance with baseline and twice weekly bilateral lower limb DVT screening ultrasound examinations will be reported.

### Current status

The trial commenced on 3 May 2010 at The Alfred Hospital, Melbourne Australia. Two interim analyses were conducted with approval by the DSMC to continue the trial without alteration to the research protocol. The target recruitment of 606 patients was achieved on 1 November 2014, making final 6-month outcomes available by May 2015.

## Conclusions

TBI is a common and devastating condition with few proven, specific therapies available. The administration of EPO has the potential to reduce neurological damage and improve neurological outcome, and is supported by a scientific rationale and laboratory data. The EPO-TBI design aims to detect an important beneficial effect of EPO on neurological function if one exists, while minimising any thrombotic risk after TBI. Application of this statistical analysis plan to the EPO-TBI trial will facilitate unbiased evaluation of these important clinical data and support confidence in the subsequent generalization of its findings. EPO-TBI aims to provide definitive guidance for clinicians regarding the true efficacy and safety of EPO in the management of TBI.

## Appendix 1: EPO-TBI outcome assessors

Heather Waddy, Australian and New Zealand Intensive Care Research Centre, Monash University, Melbourne, Australia

Marwan Al Kishi, Department of Medicine, King Abdulaziz Medical City, Riyadh, Kingdom of Saudi Arabia

Sarah Kambire, Unité de Recherche Clinique Lariboisière-Saint Louis, Assistance Publique-Hôpitaux de Paris, France

Serge Camelo, Unité de Recherche Clinique Lariboisière-Saint Louis, Assistance Publique-Hôpitaux de Paris, France

Markus Skrifvars, Intensive Care Unit, Department of Anaesthesiology and Intensive Care Medicine, Helsinki University Hospital, Helsinki, Finland

Stepani Bendel, Intensive Care Unit, Division of Anaesthesiology and Intensive Care Medicine, Kuopio University Hospital, Kuopio, Finland

Carole Schilling, Royal College of Surgeons in Ireland, Education & Research Centre, Beaumont Hospital, Dublin, Ireland

Thomas Kerz, Department of Neurosurgery, Intensive Care Therapy Unit, Universitätsmedizin, Mainz, Germany

Lynnette Murray, Australian and New Zealand Intensive Care Research Centre, Monash University, Melbourne, Australia

## Appendix 2: EPO-TBI unblinded project officer

Belinda Howe, Australian and New Zealand Intensive Care Research Centre, Monash University, Melbourne Australia

## Appendix 3: EPO-TBI management committee

Rinaldo Bellomo, Department of Intensive Care, Austin Health, Melbourne, Australia

Alistair Nichol, Department of Anaesthesia and Intensive Care Medicine, St Vincent’s University Hospital, Dublin, Ireland

Craig French, Department of Intensive Care, Western Health, Melbourne, Australia

D James Cooper, Department of Intensive Care Medicine, The Alfred, Melbourne, Australia

Olivier Huet, Intensive Care Unit, Department of Anesthesiology and Intensive Care Medicine, CHU La Cavale Blanche, Brest, France

Lorraine Little, Australian and New Zealand Intensive Care Research Centre, Monash University, Melbourne, Australia

Anne Mak, Pharmacy Department, The Alfred, Melbourne, Australia

Ville Pettilä, Intensive care Units, Division of Anaesthesiology and Intensive Care Medicine, Helsinki University Central Hospital, Helsinki, Finland

Jeffrey Presneill, Department of Intensive Care, Mater Health Services, Brisbane, Australia

Shirley Vallance, Department of Intensive Care Medicine, The Alfred, Melbourne, Australia

Dinesh Varma, Department of Radiology, The Alfred, Melbourne, Australia

Judy Wills, Department of Radiology, The Alfred, Melbourne, Australia

## Appendix 4: EPO-TBI sites, principal investigator(s) and research coordinator(s)

Auckland City Hospital, New Zealand; Colin McArthur, Yan Chen, Lynette Newby

Beaumont Hospital, Ireland; Criona Walshe, James O’Rourke, Carole Schilling,

Canberra Hospital, Australia; Imogen Mitchell, Frank Van Haren, Helen Rodgers

Christichurch Hospital, New Zealand; Seton Henderson, Jan Mehrtens, Sascha Noble

Dunedin Hospital, New Zealand; Matthew Bailey, Robyn Hutchinson, Dawn France

Gold Coast University Hospital, Australia; Brent Richards, Mandy Tallott,

Helsinki University Central Hospital, Finland; Markus Skrifvars, Heikki Vartiala, Marianne Eliasson

Hôpital Caremeau, France; Jean Yves Lefrant, Laurent Muller, Claire Roger, Christian Bengler, Pierre Barbaste

Hôpital Charles Nicolle, France; Benoit Veber, Marie Gilles-Baray, Pierre-Gildas Guitard, Helene Braud

Hôpital de Bicêtre, France; Jacques Duranteau, Anatole Harrois, Samy Figueiredo, Sophie Hamada

Hôpital Lariboisière, France; Didier Payen, Anne Claire Lukaszewicz, Charles Damoisel, Sarah Kambire

Hôpital Michallon, France; Jean François Payen, Pauline Manhes, Gilles Franconey, Perrine Boucheix

Johannes Gutenberg-Universtität, Germany; Thomas Kerz

John Hunter Hospital, Australia; Peter Harrigan, Miranda Hardie

King Abdulaziz Medical City, Kingdom of Saudi Arabia; Samir Haddad, Yaseen Arabi, Marwan Al Kishi, Ahmad Deeb, Shmeylan Al Harbi, Lolowa Al-Swaidan,Turki Al Moammar, Juliet Lingling, Shella Caliwag, Hanie Richi

Kuopio University Hospital, Finland; Stepani Bendel, Sari Rahikainen, Mikko Myllymaki

Liverpool Hospital, Australia; Victor Tam, Sharon Micallef

Nepean Hospital, Australia; Louise Cole, Leonie Weisbrodt

Royal Adelaide Hospital, Australia; Richard Strickland, Justine Rivett, Sonya Kloeden, Stephanie O’Connor

Royal Hobart Hospital, Australia; David Cooper, Richard McAllister

Royal Melbourne Hospital, Australia; Nerina Harley, Deborah Barge, Elizabeth Moore, Andrea Jordan

Royal North Shore Hospital, Australia; Simon Finfer, Elizabeth Yarad, Simon Bird, Anne O’Connor

Royal Perth Hospital, Australia; Geoffrey Dobb, Jenny Chamberlain, Michelle Barr, Elizabeth Jenkinson

Royal Prince Alfred Hospital, Australia; David Gattas, Heidi Buhr, Debra Hutch, Megan Keir

St Vincent’s Hospitaal Sydney, Australia; Priya Nair, Claire Reynolds, Serena Knowles

The Alfred, Australia; D James Cooper, Jasmin Board, Shirley Vallance, Phoebe McCracken

The Townsville Hospital, Australia; Geoffrey Gordon, Stephen Reeves

Wellington Regional Hospital, New Zealand; Richard Dinsdale, Lynn Andrews, Dianne Mackle, Sally Hurford

Westmead Hospital, Australia; Vineet Nayyar, Christina Whitehead, Jing Kong

## Appendix 5: EPO-TBI French management team

Jacques Duranteau, National Principal Investigator, Service d’Anesthésie-Réanimation, Hôpitaux universitaires Paris Sud, Assistance Publique des Hôpitaux de Paris, Hôpital de Bicêtre, Le Kremlin-Bicêtre, France

Eric Vicaut, Unité de Recherche Clinique Lariboisière-Saint Louis, Assistance Publique-Hôpitaux de Paris, France

Philippe Gallula, Pole Promotion international, Assistance Publique-Hôpitaux de Paris, France

Vidhya Raghavan, Unité de Recherche Clinique Lariboisière-Saint Louis, Assistance Publique-Hôpitaux de Paris, France

Amel Chamam, Unité de Recherche Clinique Lariboisière-Saint Louis, Assistance Publique-Hôpitaux de Paris, France

Sarah Kambire, Unité de Recherche Clinique Lariboisière-Saint Louis, Assistance Publique-Hôpitaux de Paris, France

## Electronic supplementary material

Additional file 1: **List of approving ethics committees for the EPO-TBI trial.** (PDF 317 KB)

## Abbreviations

ANZIC-RC: Australian and New Zealand Intensive Care Research Centre
CI: confidence interval
DSMC: Data and Safety Monitoring Committee
DVT: deep venous thrombosis
EPO: erythropoietin
EPO-TBI: Erythropoietin in Traumatic Brain Injury
EQ-5D: EuroQol five dimensions
GCS: Glasgow Coma Score
GOSE: Extended Glasgow Outcome Scale
SF-12: Short Form-12
TBI: traumatic brain injury.

## References

[1] Nichol AD, Higgins AM, Gabbe BJ, Murray LJ, Cooper DJ, Cameron PA (2011). Measuring functional and quality of life outcomes following major head injury: common scales and checklists. Injury.

[2] Myburgh JA, Cooper DJ, Finfer SR, Venkatesh B, Jones D, Higgins A, Bishop N, Higlett T (2008). Epidemiology and 12-month outcomes from traumatic brain injury in Australia and New Zealand. J Trauma.

[3] Moore EM, Bellomo R, Nichol AD (2011). Erythropoietin as a novel brain and kidney protective agent. Anaesth Intensive Care.

[4] Nichol AD, Cooper DJ (2009). Can we improve neurological outcomes in severe traumatic brain injury? Something old (early prophylactic hypothermia) and something new (erythropoietin). Injury.

[5] Nichol A, Little L, French C (2014). Erythropoietin for traumatic brain injury. JAMA.

[6] International Conference on Harmonisation of Technical Requirements for Registration of Pharmaceuticals for Human Use: **ICH Harmonised Tripartite Guideline: E6(R1) - Guideline for good clinical practice.** http://www.ich.org/fileadmin/Public_Web_Site/ICH_Products/Guidelines/Efficacy/E6/E6_R1_Guideline.pdf

[7] **Erythropoietin in Traumatic Brain Injury (EPO-TBI)** http://clinicaltrials.gov/show/NCT00987454

[8] Schulz KF, Altman DG, Moher D (2010). CONSORT 2010 statement: updated guidelines for reporting parallel group randomised trials. BMJ.

[9] **ICH Harmonised Tripartite Guideline: E9 - Statistical principles for clinical trials** http://www.ich.org/fileadmin/Public_Web_Site/ICH_Products/Guidelines/Efficacy/E9/Step4/E9_Guideline.pdf

[10] International Conference on Harmonisation of Technical Requirements for Registration of Pharmaceuticals for Human Use: **ICH Harmonised Tripartite Guideline: E3 - Structure and content of clinical study reports.** http://www.ich.org/fileadmin/Public_Web_Site/ICH_Products/Guidelines/Efficacy/E3/E3_Guideline.pdf 10.1111/j.1365-2125.1994.tb05705.x PMC1364893 8054244

[11] Monash University: **The Clinical Informatics and Data Management Unit (CIDMU).** http://www.med.monash.edu.au/sphpm/cidmu/

[12] Fergusson D, Aaron SD, Guyatt G, Hebert P (2002). Post-randomisation exclusions: the intention to treat principle and excluding patients from analysis. BMJ.

[13] Teasdale GM, Pettigrew LE, Wilson JT, Murray G, Jennett B (1998). Analyzing outcome of treatment of severe head injury: a review and update on advancing the use of the Glasgow Outcome Scale. J Neurotrauma.

[14] European Medicines Agency: **Guideline on adjustment for baseline covariates.** http://www.ema.europa.eu/docs/en_GB/document_library/Scientific_guideline/2013/06/WC500144946.pdf

[15] Marshall LF, Marshall SB, Klauber MR, Van Berkum CM, Eisenberg H, Jane JA, Luerssen TG, Marmarou A, Foulkes MA (1992). The diagnosis of head injury requires a classification based on computed axial tomography. J Neurotrauma.

[16] Hosmer DW, Lemeshow S, Sturdivant RX (2013). Applied Logistic Regression.

[17] Roozenbeek B, Lingsma HF, Perel P, Edwards P, Roberts I, Murray GD, Maas AI, Steyerberg EW (2011). The added value of ordinal analysis in clinical trials: an example in traumatic brain injury. Crit Care.

[18] Steyerberg EW, Mushkudiani N, Perel P, Butcher I, Lu J, McHugh GS, Murray GD, Marmarou A, Roberts I, Habbema JD, Maas AI (2008). Predicting outcome after traumatic brain injury: development and international validation of prognostic scores based on admission characteristics. PLoS Med.

[19] Cummings P (2014). Converting an adjusted odds ratio to a risk ratio will produce biased estimates. BMJ.

[20] Grant RL (2014). Converting an odds ratio to a range of plausible relative risks for better communication of research findings. BMJ.

[21] Norton EC, Miller MM, Kleinman LC (2013). Computing adjusted risk ratios and risk differences in Stata. Stata J.

[22] Ware J, Kosinski M, Keller SD (1996). A 12-Item Short-Form Health Survey: construction of scales and preliminary tests of reliability and validity. Med Care.

[23] Group EQ (1990). EuroQol - a new facility for the measurement of health-related quality of life. Health Policy.

[24] Jennison C, Turnbull BW (2000). Group Sequential Methods with Applications to Clinical Trials.

[25] The SAFE Study Investigators (2007). Saline or albumin for fluid resuscitation in patients with traumatic brain injury. N Engl J Med.

[26] Hardin JW, Hilbe JM (2012). Generalized Linear Models and Extensions.

[27] Brant R (1990). Assessing proportionality in the proportional odds model for ordinal logistic regression. Biometrics.

[28] Grambsch PM, Therneau TM (1994). Proportional hazards tests and diagnostics based on weighted residuals. Biometrika.

