# Supplementary material for: Statistical analysis plan for the Erythropoietin in Traumatic Brain Injury trial: a randomised controlled trial of erythropoietin versus placebo in moderate and severe traumatic brain injury
Source: Trials. 2014 Dec 20;15:501. doi: 10.1186/1745-6215-15-501 (PMC4414377; doi:10.1186/1745-6215-15-501)
Supplement: Supplementary file 1 — Additional file 1: List of approving ethics committees for the EPO-TBI trial. (PDF 317 KB) [file 13063_2014_2416_MOESM1_ESM.pdf]

Project Title: Erythropoietin in Traumatic Brain Injury
List of Participating Centres and responsible Ethics Committees

Country / Site	Ethics Committee	Reference Number
**Australia**		
The Canberra Hospital	ACT Health Human Research Ethics Committee	ETH 11.09.1004
John Hunter Hospital	Sydney West Area Service HREC (Nepean Campus)	09-49
Liverpool Hospital	Sydney West Area Service HREC (Nepean Campus)	09-49
Nepean Hospital (*Lead Site*)	Sydney West Area Service HREC (Nepean Campus)	09-49
Royal North Shore Hospital	Sydney West Area Service HREC (Nepean Campus)	09-49
Royal Prince Alfred Hospital	Sydney West Area Service HREC (Nepean Campus)	09-49
St Vincent's Sydney	Sydney West Area Service HREC (Nepean Campus)	09-49
Westmead Hospital	Sydney West Area Service HREC (Nepean Campus)	09-49
Gold Coast Hospital	Gold Coast Health Services District Ethics Committee	HREC09QG-82
The Townsville Hospital	Townsville Health Service District Ethics Committee	HREC09QTHS120
Royal Adelaide Hospital	Royal Adelaide Hospital HREC	100812
Royal Hobart Hospital	Human Research Ethics Committee (Tasmania)	H0011010
Alfred Hospital	Alfred Hospital Ethics Committee	156/09
The Royal Melbourne Hospital	Melbourne Health HREC	2009-183
Royal Perth Hospital	Royal Perth Hospital Ethics Committee	EC 2010/016
**New Zealand**		
Auckland City Hospital	Multi-region Ethics Committee	MEC/10/01/006
Christchurch Hospital	Multi-region Ethics Committee	MEC/10/01/006
Wellington Regional Hospital	Multi-region Ethics Committee	MEC/10/01/006
Dunedin Hospital	Multi-region Ethics Committee	MEC/10/01/006
**Kingdom of Saudi Arabia**		
King Abdulaziz Medical City	Institutional; Review Board. National Guard Health Affairs - King Abdulaziz Medical City	RC10/063
**Finland**		
Helsinki University Central Hospital	Helsingin Ja Uudenmaan Sairaanhoitopuri Helsingfors OCH Nylands Sjukvardsdistrikt	295.13.03.02.2011
Kuopio University Hospital	Helsingin Ja Uudenmaan Sairaanhoitopuri Helsingfors OCH Nylands Sjukvardsdistrikt	295.13.03.02.2011

Country / Site	Ethics Committee	Reference Number
**France**		
Hôpital de Bicêtre	Comite De Protection Des Personne – Ile de France 1	2012-mars-12863
Hôpital Lariboisière	Comite De Protection Des Personne – Ile de France 1	2012-mars-12863
Hôpital Michallon	Comite De Protection Des Personne – Ile de France 1	2012-mars-12863
Hôpital Caremeau	Comite De Protection Des Personne – Ile de France 1	2012-mars-12863
Hôpital Charles-Nicolle	Comite De Protection Des Personne – Ile de France 1	2012-mars-12863
**Germany**		
Johannes Gutenberg-Universtität	Ethik Kommission der Landesaerztekammer Rheinland – Pfalz	837.383.12 (8484-A)
**Ireland**		
Beaumont Hospital	Beaumont Hospital Ethics (Medical Research) Committee	13.04
